# A case of transverse colon cancer with a large liver abscess that could be treated with a radical operation after infection control

**DOI:** 10.1016/j.ijscr.2020.10.122

**Published:** 2020-11-04

**Authors:** Hideki Kogo, Kazuhito Yamamoto, Hiroshi Yoshida

**Affiliations:** a Department of Surgery, Nippon Medical School Tama-Nagayama Hospital, Tokyo, Japan; b Department of Surgery, Kitamurayama Hospital, Yamagata, Japan; c Department of Gastrointestinal and Hepato-Biliary-Pancreatic Surgery, Nippon Medical School, Tokyo, Japan

**Keywords:** Advanced colorectal cancer, Case report, Liver abscess, Percutaneous transhepatic abscess drainage (PTAD)

## Abstract

**Introduction:**

Advanced colorectal cancer is known to be associated with liver abscesses. A strategy to treat liver abscesses as early as possible is necessary to ensure high surgical curability before the cancer progresses and to prevent worsening nutritional status of the patient during surgery.

**Presentation of case:**

An 82-year-old woman was hospitalized due to fever. A 10 cm in diameter liver abscess and advanced colon cancer were diagnosed. After conservative treatment of the liver abscess with antibiotics and puncture drainage, colorectal cancer surgery was performed on a standby basis.

**Discussion:**

We propose that empiric antibiotic therapy should be administered early in cases of liver abscesses that may require early colorectal surgery. Abscess drainage should be performed promptly if the abscess is of a size that can be punctured easily.

**Conclusion:**

In patients with advanced colorectal cancer complicated by liver abscesses that required early surgery, prompt drainage of the liver abscesses is mandatory.

## Highlights

• Advanced colorectal cancer is known to be associated with liver abscesses.
• A strategy to treat liver abscesses as early as possible is necessary to prevent worsening nutritional status of the patient for surgery.
• Prompt drainage of liver abscesses is essential in patients with colorectal cancer complicated by liver abscesses that require early surgery.

## 1 Introduction

Advanced colorectal cancer is known to be associated with liver abscesses [[1], [2], [3], [4], [5]]. Several reports exist of latent colorectal cancer in patients with liver abscesses. Therefore, if a liver abscess is found, close examination for gastrointestinal disease is recommended [6]. If colorectal cancer is identified, treatment must be initiated as early as possible, especially for advanced cancer. In this case, treatment of colorectal cancer should be preceded by treatment of liver abscesses. However, treatment of liver abscesses is known to take a long time. If surgery is being planned for colorectal cancer, a lot of time cannot be taken to treat the liver abscesses. A strategy to treat liver abscesses as early as possible is necessary to ensure high surgical curability before the cancer progresses and to prevent worsening nutritional status of the patient during surgery. We report a case of advanced transverse colon cancer with a large liver abscess that was treated successfully with radical therapy after infection control.

## 2 Case description

**Fig. 1:**
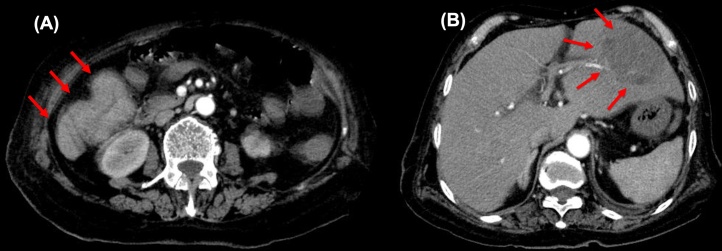
Enhanced CT scan demonstrates a transverse colon tumor (**A**) with liver abscess (**B**).

**Fig. 2:**
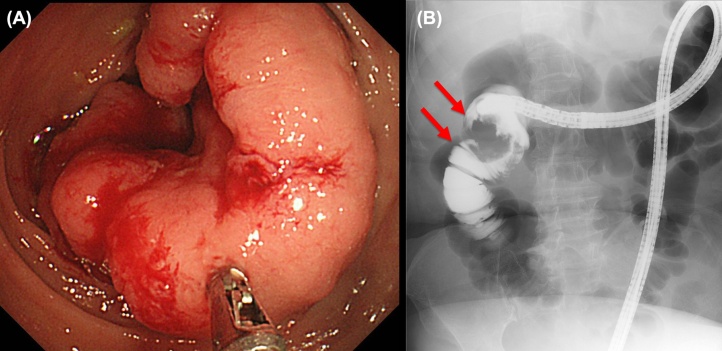
Type 2 tumor was identified in the transverse colon. (**A**) Lower gastrointestinal endoscopy. (**B**) Gastrointestinal endoscopy angiography. Pathologic finding revealed well-differentiated adenocarcinoma.

An 82-year-old woman with hypertension was hospitalized because of fever (39 °C). The abdomen was tender, without signs of peritonitis. Blood tests revealed an increased white blood cell count of 15,790/μl, C-reactive protein 24.52 mg/dL, hemoglobin 8.2 mg/dL, carcinoembryonic antigen 8.9 mg/dL, and CA19-9 level 22.4 mg/dL. Computed tomography (CT) revealed a 10 cm in diameter liver abscess and inflammation of the colon (Fig. 1). Lower gastrointestinal endoscopy revealed a type 2 tumor in the transverse colon. Pathology revealed well-differentiated adenocarcinoma. The endoscope was passable, but the tract was becoming narrower (Fig. 2). The patient required early surgery and we performed preoperative examination in parallel with treatment of the liver abscess. Furthermore, it was necessary to improve her general condition for the operation.

At diagnosis of the liver abscess, tazobactam/piperacillin (TAZ/PIPC) antibiotics were started empirically. *Klebsiella pneumoniae* was detected in the admission blood culture, and we confirmed it to be susceptible to TAZ/PIPC.

**Fig. 3:**
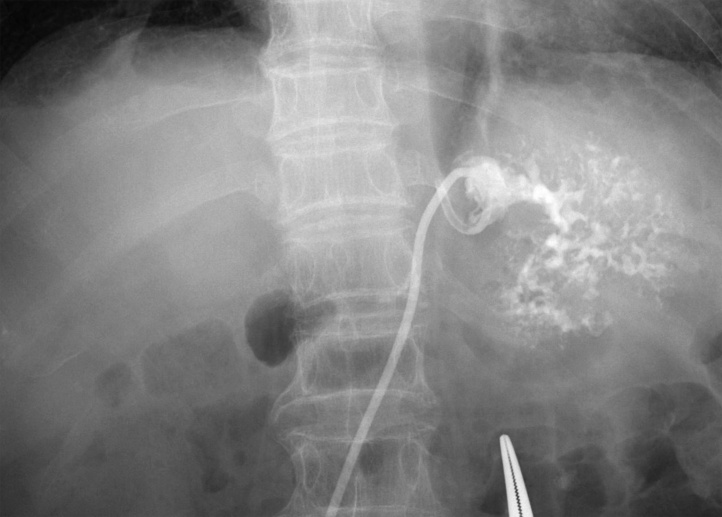
Percutaneous transhepatic drainage of the liver abscess (7 French pigtail catheter) was performed 2 weeks after the start of treatment.

We considered performing percutaneous transhepatic abscess drainage (PTAD), but decided against this initially because it was deemed to be less effective to drain due to the presence of a septum in the abscess [7]. However, approximately 2 weeks after admission, the inflammatory response was elevated again and fever also was observed. A CT scan showed that the liver abscess was slightly enlarged without shrinkage. PTAD was performed because antibiotic treatment alone was not sufficient to cure the liver abscess (Fig. 3). A white viscous pus discharge was observed, and *K. pneumoniae* was detected in the blood and discharge cultures. After drainage, the fever resolved and the inflammatory response improved markedly.

**Fig. 4:**
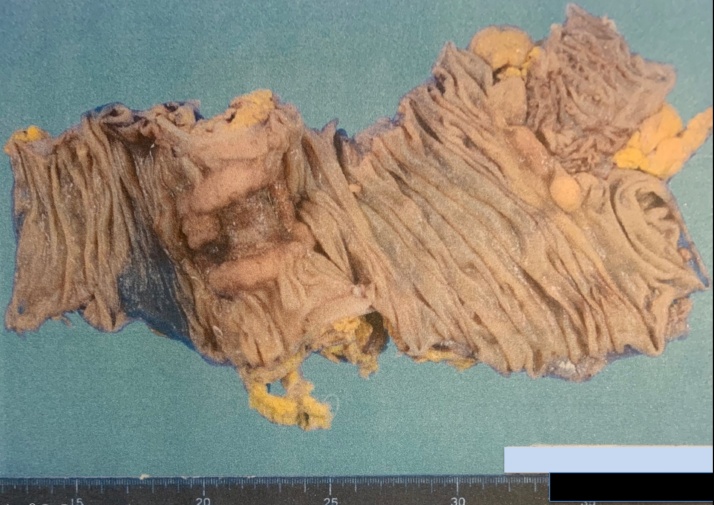
Resected specimen. T, Type2; 50 × 45 mm, tub1, stage pT3, INFb, ly1a, v0, Pn0, pN0, pPM0, pDM0, pStageIIa.

TAZ/PIPC was administered for a total of 28 days. The abscess drainage tube showed a very small amount of drainage with improvement in inflammation and the patient's general condition. Contrast studies were done at 12 days after insertion and the tube was removed after confirmation of abscess resolution. Approximately 4 weeks after the start of treatment, the patient underwent standby surgery for transverse colon cancer (hepatic flexure; Fig. 4), consisting of laparoscopic converted to open right hemi-colectomy (D3). Operative time was 195 min and estimated blood loss was minimal. The procedure was converted due to strong adhesion of the tumor to the duodenum.

The patient progressed well postoperatively, except for a minor postoperative wound infection, and was discharged approximately 20 days postoperatively.

No cancer/hepatic abscess recurrence was noted at followup approximately 1 year postoperatively. The case has been reported in line with the SCARE criteria [8].

## 3 Discussion

The clinical presentation of liver abscess generally includes fever, right abdominal pain, hepatomegaly, and tenderness, and its development often occurs in multiple areas in bacterial cases and in a single right lobe in amoebic cases [9,10]. Risk factors include diabetes mellitus, liver cirrhosis, cardiac disease, lung disease, malignancy, anticancer drugs, steroid administration, a history of foreign travel, male homosexuals, heavy alcohol consumption, and caries [9,10].

The classification of liver abscesses can be divided broadly into bacterial and amoebic liver abscesses. Bacterial liver abscesses are characterized by the following [9,10]. The epidemiology is most commonly in middle-aged and older patients (>50 years old) with no difference in sex incidence. The infection route is transbiliary, transportal, transarterial, direct, traumatic, and medico-genic/idiopathic [11]. Causative agents include *Escherichia coli*, *Klebsiella*, *Streptococcus anginosus* group, *Enterococcus*, and *Bacteroides* [12].

With regard to the route of infection resulting in the development of liver abscesses from colon cancer, it is believed that growth of the colon tumor causes destruction of the intestinal wall and formation of microabscesses around the tumor, leading to a transmural bacterial infection. Bacterial invasion into the portal system, with subsequent hematogenous spread to the liver will lead to liver abscess [2,5].

The following methods are recommended for initial antibiotic treatment.

The maximum dose of sulbactam sodium/ampicillin sodium, TAZ/PIPC, ceftriaxone, metronidazole (MNZ), and so forth should be administered. If fever/inflammation does not improve after 2–3 days, consider changing to ciprofloxacin, pazufloxacin, and MNZ or to carbapenems [13,14]. Change antibiotics as soon as the causative organism is identified.

This case presented as a transbiliary bacterial liver abscess in a carrier cancer patient. Blood and abscess culture results confirmed that the causative organism was *K. pneumoniae*. Treatment of liver abscesses often is relatively time-consuming. Therefore, it is not possible to start colon cancer treatment immediately. Liver abscesses are treated with antibiotics for a minimum of 2–4 weeks, and often require 6 weeks of treatment [15].

However, in cases of colon cancer, especially if the cancer is advanced and on the verge of becoming subileus, as in our case, it is not possible to wait for treatment. Our patient was hospitalized and underwent surgery approximately 1 month after diagnosis. However, retrospectively, the preoperative wait time could have been shortened if we had performed PTAD earlier.

The main treatments of liver abscess are with antibiotics and drainage [16]. Some studies have reported on partial hepatectomy for liver abscesses or surgical drainage, but these can be difficult to perform in general [1,9]. Antibiotic therapy is the mainstay of treatment, while drainage is used for treatment and diagnosis. It is important to perform these two therapies as soon as possible to confirm the causative organisms and their susceptibility to antibiotics by culture, and to suppress inflammation by drainage to prevent worsening of the nutritional status of the patient (Table 1). Although it is basic to treat amoebic liver abscesses without drainage [9], reports have recommended drainage in cases where the diagnosis is urgent [17]. Also, even if drainage seems less effective because of the presence of a septum in the abscess, if there is a fluid retention, the abscess should be punctured. This is because drainage is useful not only for treatment but also for diagnosis and identification of the causative organism. We propose that empiric antibiotics should be administered early in cases of liver abscesses that may require early surgery. Abscess drainage should be performed promptly if the abscess is of a size that can be easily punctured.

**Table 1:** A therapeutic strategy for advanced colorectal cancer with concomitant liver abscesses.

## 4 Conclusion

In patients with advanced colorectal cancer complicated by liver abscesses that required early surgery, prompt drainage of the liver abscesses is mandatory.

## Declaration of Competing Interest

The authors report no declarations of interest.

## Funding

None.

## Ethical approval

Not applicable.

## Consent

Informed consent was obtained from the patient for the publication of this case report and accompanying images.

## Author contribution

HK: Drafted the manuscript.

HK and KY performed the operation.

HK, KY, and HY: Revised the manuscript.

HK, KY, and HY: Read and approved the final manuscript.

## Registration of research studies

Not applicable.

## Guarantor

On the behalf of all author I am the guarantor. Hideki Kogo.

## Provenance and peer review

Not commissioned, externally peer-reviewed.
